# *RAD51* and *RAD50* genetic polymorphisms from homologous recombination repair pathway are associated with disease outcomes and organ toxicities in AML

**DOI:** 10.1007/s44313-024-00033-7

**Published:** 2024-12-30

**Authors:** Alireza Mohseni, Gholamreza Toogeh, Shahrbano Rostami, Mohammad Faranoush, Mohammad Jafar Sharifi

**Affiliations:** 1 Thalassemia Research Center, Hemoglobinopthy Institute, Mazandaran University of Medical Sciences, Sari, Iran; 2 Department of Internal Medicine, School of Medicine, Tehran University of Medical Sciences, Tehran, Iran; 3 Tehran University of Medical Sciences, Tehran, Iran; 4 Pediatric Growth and Development Research Center, Iran University of Medical Sciences, Tehran, Iran; 5 Division of Laboratory Hematology and Blood Banking, Department of Medical Laboratory Sciences, School of Paramedical Sciences, Shiraz University of Medical Sciences, Meshkin Fam Street, P.O. Box, Shiraz, 71345-1744 Iran

**Keywords:** Acute Myeloid Leukemia, Outcomes, Organ toxicity, Chemotherapy, Pharmacogenetic

## Abstract

**Background:**

Acute myeloid leukemia (AML) is a heterogeneous malignancy that responds to various therapies. The sensitivity of leukemia cells to chemotherapy is affected by the DNA damage response (DDR). In this study, we examined the association between *RAD51* rs1801320, *XRCC3* rs861539, *NBS1* rs1805794, *MRE11* rs569143, and *RAD50* rs2299014 variants of the homologous recombination repair (HRR) pathway and AML outcomes.

**Material and methods:**

PCR–RFLP was applied for the genotyping of 67 newly diagnosed cases. We performed Sanger sequencing to confirm the results of RFLP genotyping. Outcomes and organ toxicities were collected and χ^2^ testing was performed for association analysis.

**Results:**

*RAD50* variant allele carriers were protected from renal and hepatic toxicities (*p* = 0.024 and *p* = 0.045, respectively), and were associated with resistant disease (*p* = 0.001). *RAD51* variant alleles were protected from liver toxicity (*p* = 0.031) and correlated with disease resistance (*p* = 0.012).

**Conclusion:**

*RAD50* rs2299014 and *RAD51* rs1801320 polymorphisms may be useful for drug adjustment in AML.

**Supplementary Information:**

The online version contains supplementary material available at 10.1007/s44313-024-00033-7.

## Introduction

Acute myeloid leukemia (AML) is a disease with heterogeneous pathophysiology and treatment response. It is the most common form of leukemia among adults. The prognosis and response to treatment depend on patient age and cytogenetic/molecular risk stratification [1]

]. Significant progress has been achieved in drug development [2]. Improvements in disease outcomes have been associated with increased attention being paid to the side effects of chemotherapy. Unlike prognostic and predictive biomarkers for disease outcomes, definitive biomarkers for predicting side effects and organ toxicities in AML remain unidentified [3]. Genetic variants play an essential role in dose adjustment and personalized medicine for malignancies [4–6]. While there are well-known examples of pharmacogenetic biomarkers of acute lymphoblastic leukemia (ALL), further investigation of AML is required. Chemotherapeutic agents kill leukemic cells through genotoxic stress and apoptosis [7, 8]. DNA damage response (DDR) proficiency could alter the leukemic cell susceptibility to chemotherapeutic agents [9, 10]. Considering previous studies which demonstrated the potential pharmacogenetic roles for genetic variants of DDR pathways in AML [11–14], we also investigate the association of *RAD51* rs1801320, *XRCC3* rs861539*, NBS1* rs1805794*, MRE11* rs569143, *RAD50* rs2299014 variants with AML outcomes. The selected genes belong to the homologous recombination repair (HRR) pathway, which plays an important role in DDR systems. We selected these genetic variants based on literature review and their association with cancer outcomes.

## Materials & methods

We included 67 adult patients who were newly diagnosed with de novo AML at Shariati and Imam Khomeini hospitals between 2014 and 2016. Patients with acute promyelocytic leukemia (APL) were excluded because of their different prognoses and treatment protocols. At the time of diagnosis, mononuclear cells from blood specimens or bone marrow aspirates were collected and stored at − 70 °C. We obtained informed consent from all patients prior to sample collection. Additionally, we excluded patients with any history of malignancy. Morphological examination and standard multicolor flow cytometry were performed for all patients, and the final diagnosis was made according to the WHO Health Organization 2008 classification criteria [15]. Karyotype analysis was performed on 43 participants only. The patient characteristics are summarized in Table 1. All patients received remission induction chemotherapy with daunorubicin (45 mg/m2 per day for 3 days) and standard-dose cytosine arabinoside (200 mg/m2 per day for 7 days) with or without recombinant human granulocyte colony-stimulating factor.

**Table 1:** Patients characteristics data. Cytogenetic risk classification based on WHO guidelines was provided for only 43 patients with cytogenetic results

Patient numbers		67
Age (years), median (range)		41(15–65)
Gender, no (%)	Male	38(56.7)
	Female	29(43.3)
Leukocyte count, 10^9^/L, median (range)		29,900 (400–215,300)
Blast (%); peripheral blood, median (range)		39(5–39)
Blast (%); Bone Marrow, median (range)		80 (29–98)
Hemoglobin g/dL, median (range)		8.9 (3.3–9.7)
Platelet,10^9^/L, median (range)		47,000 (1,000–27,000,0)
**Low-risk karyotype;** Inv16, t(8;21)		6 (9%)
**High-risk Karyotype;** del5q, -5, -7, 3q abnormalities and complex		5 (7.5%)
**Intermediate-risk karyotype;** All others		32 (83.5%)

### Genotyping

Genomic DNA was extracted using a QIAmp DNA Blood Mini Kit (Qiagen, Hilden, Germany). All the target sequences containing polymorphic variants were amplified using specific primers. Agarose gel electrophoresis was used to characterize the PCR products and verify the PCR optimization. Restriction enzymes (RFLP) were used for genotyping. *RAD51* rs1801320), *XRCC3* rs861539, *NBS1* rs1805794, *MRE11* rs569143, and *RAD50* rs2299014 genetic variants were characterized using BstNI, NIaIII, HinfI, and HinP1I restriction enzymes, respectively. Ten PCR products from each polymorphic variant were randomly selected and subjected to Sanger sequencing. Complete concordance between PCR–RFLP and DNA sequencing results was obtained.

### Response to induction therapy & related toxicities

The European Leukemia Net recommendations for response assessment were applied [16]. The treatment response rate was assessed on day 28 after initiating induction therapy. The ELN guidelines define complete remission (CR) as bone marrow blasts less than 5%, absence of blasts with Auer rods, absence of extramedullary disease, absolute neutrophil count > 1.0 × 10^9^/L (1000/μl), platelet count > 100 × 10^9^/L (100,000/μl) independent of red cell transfusions. Partial remission (PR) was defined as bone marrow blast percentage of 5%–25%, accompanied by all other hematologic criteria for CR. Patients with CR and and those with PR were categorized as responders, whereas those with treatment failure were categorized as non-responders. On average, our cohort was followed-up for 14 months to detect disease relapse. Disease relapse in patients with CR is defined as the reappearance of blasts in the peripheral blood, > 5% bone marrow blasts, or the development of extramedullary disease. Studied patients were evaluated for induction therapy-related toxicities according to Common Terminology Criteria for Adverse Events version 3.0 (CTCAE v3.0)[17]. Liver, renal, lung, metabolic, and gastrointestinal (GI) organ damage was determined based on clinical and laboratory findings. Based on toxicity severity, four grades of organ damage are defined by CTCAE v3.0. We classified the patients into toxic (grades 1–4) and nontoxic (grade 0) groups for better statistical analysis.

We used the chi-square (χ^2^) test to determine if there is an association between two categorical variables: disease outcome and organ toxicities associations with genetic variants. A *p*-value of less than 0.05 is considered to indicate a significant statistical correlation.

## Results

### Genetic variant frequencies

Genotype frequencies in studied population were; *RAD50* rs2299014, 39 GG (58.2%0), 18 GT (26.9%) and 10 TT (14.9%); *MRE11* rs569143, 26 CC (38.8%), 27 CG (40.3%) and 14 GG (20.9%); *NBS1* rs1805794, 25 CC (37.3%), 32 CG (47.8%) and 10 GG (14.9%); *XRCC3* rs861539, 33 CC (49.3), 25 CT (37.3%) and 9 TT (13.4%); *RAD51* rs1801320, 37 GG (55.2%), 20 GC (29.9%) and 10 CC (14.9%). There were no deviations from Hardy–Weinberg equilibrium for each polymorphism (*P* > 0.05).

### Correlations with disease outcomes

For χ^2^ testing, we put the wild-type homozygous genotype on one side and the sum of heterozygous and variant homozygous genotypes on the other side. Correlation of outcome of interest with genotypes was analyzed. The association of resistance to induction therapy and genetic variants is shown in Table 2. As demonstrated in Table 2, *RAD50* rs2299014 (*p* < 0.01) and *RAD51* rs1801320 (*p* = 0.012) variant alleles were associated with resistant disease.

**Table 2:** Correlation of polymorphisms with resistance to induction therapy

Polymorphism (n)	Resistance to induction Therapy (%)	OR (95% CI)	*P.value*
RAD51 rs1801320			
GG (37)	4 (26.7)	Reference	
GC + CC (30)	11(73.3)	4.77(1.33–17.11)	*0.012†*
XRCC3 rs861539			
CC (33)	8 (53.3)	Reference	
CT + TT (34)	7(46.7)	0.810(0.256–2.56)	0.72
NBS1 rs1805794			
CC (25)	3 (20.0)	Reference	
CG + GG (42)	12 (80.0)	2.93(0.738–11.65)	0.116
MRE11 rs569143			
CC(26)	6 (40.0)	Reference	
CG + GG(41)	9 (60.0)	0.938 (0.290–3.03)	0.914
RAD50 rs2299014			
GG (39)	3 (20.0)	Reference	
GT + TT (28)	12 (80.0)	9.00(2.22–36.33)	0.001†

^†^ Statistically significant difference

A χ^2^ analysis was performed Concerning the possible effect of other variables like age, morphologic subgroups, and WBC count on response to induction therapy. There were no correlations with morphologic subgroups, WBC count, and age (*p* = 0.612, *p* = 0.725, and *p* = 0.296, respectively). Finally, genetic variants were considered as independent prognostic factors with no need for multiple regression analysis to adjust the effect of other variables.

### Correlations with organ toxicities

#### Renal toxicity

*RAD50* rs2299014 [GG vs GT + TT, OR(95%CI); 0.298(0.102–0.872), *p* = 0.024]. No correlations were detected for the other polymorphisms.

#### Hepatotoxicity

*RAD51* rs1801320 [GG vs GC + CC OR(95%CI); 0.315 (0.109–0.912), *p* = 0.031], *RAD50* rs2299014 [GG vs GT + TT OR(95%CI); 0.346 (0.121–0.991), *p* = 0.045]. No associations were detected between other organ toxicities (GI, lung, and metabolic) and the genetic variants (data not shown).

## Discussion

Our findings revealed the association of two polymorphic alleles, *RAD50* rs2299014 and *RAD51* rs1801320, with clinical outcomes of AML. Genetic variants of DDR proteins, multidrug resistance-related proteins, and glutathione *S*-transferases are suspected candidate**s** for drug resistance in AML [18]. However, promising results have been achieved using inhibitors of DDR for AML therapy, emphasizing the important role of the DDR system in AML chemosensitivity [19]. *RAD50* and *RAD51* are involved in homologous recombination and important for genomic stability [20]. Our results revealed that *RAD51* rs1801320 variant allele carriers (GC + CC) were associated with resistance to induction therapy (OR: 4.7, *p* = 0.012). Similar correlations were also detected by Liu et al. in a cohort of patients with AML [21]. However, these associations were not observed in other associations [11, 22, 23].

The substitution of G to C in *RAD51* rs1801320 (G135C) occurs in the promoter region and is associated with increased gene expression and elevated protein levels and activity [24]. Interestingly, subjects with variant alleles (GC + CC) were protected from liver toxicity compared with those with wild-type alleles (GG) (OR: 0.315, *p* = 0.031).

There are limited and conflicting data regarding the correlation between hepatotoxicity and *RAD51* rs1801320 polymorphism. Increased RAD51 activity may result in increased resistance to the genotoxic effects of chemotherapy in leukemic and nonmalignant cells. This hypothesis should be examined using an in vitro model of cells harboring the wild-type and variant alleles.

Park cohort of AML patients, a correlation of poor overall survival (OS) probability with overexpression of the *RAD51* gene was detected of S.Park et al. [25]. shRNA targeting sensitized T lymphoblastic leukemia cells to chemotherapy and demonstrated the central role of *RAD51* in chemosensitivity [26]. *RAD51* is a key element of the HRR pathway, and the rs1801320 genetic variant is a well-established risk factor for therapy-related AML [27].

*RAD50* rs2299014 variant allele carriers (GT + TT) were protected from renal and hepatic toxicities (OR, 0.298; *p* = 0.024 OR; 0.346, *p* = 0.045, respectively). A significant odds ratio for the correlation of variant genotypes (GT + TT) with resistant disease was identified (OR, 9.0; *p* = 0.001). To the best of our knowledge, there have been no reports regarding the association between *RAD50* rs2299014 genetic polymorphisms and clinical outcomes in AML. Further studies are required to confirm these findings. Since *RAD50* rs2299014 is an intronic variant, it may alter transcript splicing and protein quantity in cells. The possibility of linkage disequilibrium (LD) with other significant variants should also be considered.

Leukemic and normal somatic cells could both benefit from the increased efficacy of the HRR pathway to repair genotoxic damage during chemotherapy for AML. The overall effects of these abilities would be reduced in resistant diseases with fewer organ toxicities. The *RAD51* rs1801320 (G135C) variant is associated with increased expression of the *RAD51* protein and robust DNA damage responses. However, further in vitro investigations should be performed on the *RAD50* rs2299014 polymorphism to reveal similar effects. It could be presumed that normal cells from vital organs, such as the liver and kidneys, could rescue themselves from the genotoxic effects of AML chemotherapy if they had a more efficient DNA damage response.

It should be kept in mind that the interaction of other biological pathways, the specific sensitivity of each organ to therapeutic regimens, and modifications of treatments could determine the severity of organ toxicities. Therefore, these factors should be considered in future pharmacogenetic studies. To resolve the association of organ toxicities with genetic variants, more robust genotyping techniques such as high-throughput sequencing, prospective studies, well-documented clinical findings, more homogeneous care, and therapeutic regimens should be applied to leukemia cells. Normal somatic cells could benefit from the increased efficacy of the HRR pathway to repair their genotoxic damages during chemotherapy for AML. The net effects of these abilities include resistance to diseases and fewer organ toxicities.

Other studies have not investigated the *MRE11* rs569143 polymorphism. No association with clinical outcomes was detected by our study for *MRE11* rs569143 polymorphism. Other genetic variants of the HRR pathway (*NBS1* and *XRCC3*) did not correlate with clinical outcomes or organ-related toxicities in the present study. However, other groups have reported some correlations [21, 28, 29]. In addition to HRR polymorphisms, HRR pathway gene expression scores can be used as independent prognostic factors in AML [30].

Our study has a small sample size and incomplete cytogenetic results. Cytogenetic abnormalities and somatic mutations are the most important prognostic factors in AML and a predictive value of new biomarkers should be adjusted for these variables [1, 3, 16]. This association may resolve after adjusting for abnormal cytogenetic or somatic mutations. Another approach to solve this problem is to examine possible associations in a selected group of patients. For example, it is possible to study subjects with normal cytogenetics, which include half of the AML patients [13, 22].

Therefore, patients in our study were selected from the non-APL AMLs group. Patients with APL undergo different therapeutic protocols and have different clinical outcomes than those without APL. Patients with therapy-related AMLs were also excluded. Pediatric and elderly patients were excluded from the study. These are independent prognostic factors for AML and their determining effects are limited by appropriate patient selection. The patients in the present study had similar genetic backgrounds, which plays an important role in association studies.

With the advent of high-throughput technologies such as next-generation sequencing (NGS), candidate gene-related studies are expected to become limited. However, these approaches remain important research tools for low-resource centers, and the resulting findings should be considered. The ultimate goal of precision medicine and pharmacogenetic studies is to adjust the medication for each patient based on predictive biomarkers. Our results may be used as confirmatory (for *RAD51*) or primary (for *RAD50*) data in personalized medical settings.

## Conclusion

Overall, our results show a significant correlation between *RAD50* rs2299014 genetic polymorphism and AML outcomes for the first time. Consistent with previous findings, *RAD51* rs1801320 was correlated with disease resistance in our study. If replicated in other groups, these findings can be used for treatment adjustments.

## Supplementary Information

Supplementary Material 1.

## Data Availability

No datasets were generated or analysed during the current study.

## Availability of data and materials

All data generated or analyzed during this study are included in this manuscript.
