# Supplementary material for: RAD51 and RAD50 genetic polymorphisms from homologous recombination repair pathway are associated with disease outcomes and organ toxicities in AML
Source: Blood Res. 2024 Dec 30;59(1):46. doi: 10.1007/s44313-024-00033-7 (PMC11685162; doi:10.1007/s44313-024-00033-7)
Supplement: Supplementary file 1 — Supplementary Material 1. [file 44313_2024_33_MOESM1_ESM.docx]

| **Association of genetic variants with renal toxicity.** | | | |
| --- | --- | --- | --- |
| ***P.value*** | **OR (95% CI)** | **Renal toxicity, N (%)** | **Polymorphism (n)** |
| **RAD51 rs1801320** | | | |
| **0.457** | **Reference**  **1.49(0.519-4.27)** | **13 (61.9)**  **8 (38.1)** | **GG (37)**  **GC+CC (30)** |
| **XRCC3 rs861539** | | | |
| **0.479** | **Reference**  **0.688(0.243-1.94)** | **9 (42.9)**  **12(57.1)** | **CC (33)**  **CT+TT (34)** |
| **NBS1 rs1805794** | | | |
| **0.567** | **Reference**  **0.568 (0.187-1.73)** | **6 (28.6)**  **15 (71.4)** | **CC (25)**  **CG+GG (42)** |
| **MRE11 rs569143** | | | |
| **0.936** | **Reference**  **0.957 (0.331-2.72)** | **8 (38.1)**  **13 (61.9)** | **CC(26)**  **CG+GG(41)** |
| **RAD50 rs2299014** | | | |
| **0.024†** | **Reference**  **0.298 (0.102-0.872)** | **8 (38.1)**  **13 (61.9)** | **GG (39)**  **GT+TT (28)** |
| **† *Statistically significant difference*** | | | |

| **Association of genetic variants with liver toxicity.** | | | |
| --- | --- | --- | --- |
| ***P.value*** | **OR (95% CI)** | **Liver toxicity, N (%)** | **Polymorphism (n)** |
| **RAD51 rs1801320** | | | |
| **0.031†** | **Reference**  **0.315 (0.109-0.912)** | **8 (36.4)**  **14 (63.6)** | **GG (37)**  **GC+CC (30)** |
| **XRCC3 rs861539** | | | |
| **0.575** | **Reference**  **1.37 (0.493-3.81)** | **12 (54.5)**  **10 (45.5)** | **CC (33)**  **CT+TT (34)** |
| **NBS1 rs1805794** | | | |
| **0.516** | **Reference**  **0.700 (0.238-2.05)** | **7 (31.8)**  **15 (88.2)** | **CC (25)**  **CG+GG (42)** |
| **MRE11 rs569143** | | | |
| **0.412** | **Reference**  **0.629 (0.218-1.87)** | **7 (31.8)**  **15 (88.2)** | **CC(26)**  **CG+GG(41)** |
| **RAD50 rs2299014** | | | |
| **0.045†** | **Reference**  **0.346 (0.121-0.991)** | **9 (40.9)**  **13 (59.1)** | **GG (39)**  **GT+TT (28)** |
| **† *Statistically significant difference*** | | | |

| **Association of genetic variants with Nausea.** | | | |
| --- | --- | --- | --- |
| ***P.value*** | **OR (95% CI)** | **Nausea, n (%)** | **Polymorphism (n)** |
| **RAD51 rs1801320** | | | |
| **0.383** | **Reference**  **0.650 (0.247-1.71)** | **17 (50.0)**  **17 (50.0)** | **GG (37)**  **GC+CC (30)** |
| **XRCC3 rs861539** | | | |
| **0.540** | **Reference**  **1.35 (0.517-3.52)** | **18 (52.9)**  **16 (47.1)** | **CC (33)**  **CT+TT (34)** |
| **NBS1 rs1805794** | | | |
| **0.729** | **Reference**  **0.952 (0.311-2.26)** | **12 (35.3)**  **22 (64.7)** | **CC (25)**  **CG+GG (42)** |
| **MRE11 rs569143** | | | |
| **0.412** | **Reference**  **0.956 (0.356-2.54)** | **13 (38.2)**  **21 (61.8)** | **CC(26)**  **CG+GG(41)** |
| **RAD50 rs2299014** | | | |
| **0.579** | **Reference**  **1.34 (0.508-3.56)** | **21 (61.8)**  **13 (38.2)** | **GG (39)**  **GT+TT (28)** |
| **† *Statistically significant difference*** | | | |

| **Association of genetic variants with Vomiting.** | | | |
| --- | --- | --- | --- |
| ***P.value*** | **OR (95% CI)** | **Vomiting, n (%)** | **Polymorphism (n)** |
| **RAD51 rs1801320** | | | |
| **0.466** | **Reference**  **0.696 (0.262-1.84)** | **14 (50.0)**  **14 (50.0)** | **GG (37)**  **GC+CC (30)** |
| **XRCC3 rs861539** | | | |
| **0.643** | **Reference**  **0.643 (0.242-1.71)** | **12 (42.9)**  **16 (57.1)** | **CC (33)**  **CT+TT (34)** |
| **NBS1 rs1805794** | | | |
| **0.210** | **Reference**  **0.518 (0.184-1.45)** | **8 (28.6)**  **20 (71.4)** | **CC (25)**  **CG+GG (42)** |
| **MRE11 rs569143** | | | |
| **0.946** | **Reference**  **1.03 (0.383-2.80)** | **11 (39.3)**  **17 (60.7)** | **CC(26)**  **CG+GG(41)** |
| **RAD50 rs2299014** | | | |
| **0.393** | **Reference**  **1.54 (0.569-4.18)** | **18 (64.3)**  **10 (35.7)** | **GG (39)**  **GT+TT (28)** |
| **† *Statistically significant difference*** | | | |

| **Association of genetic variants with Diarrhea .** | | | |
| --- | --- | --- | --- |
| ***P.value*** | **OR (95% CI)** | **Diarrhea, n (%)** | **Polymorphism (n)** |
| **RAD51 rs1801320** | | | |
| **0.961** | **Reference**  **0.968 (0.256-3.54)** | **6 (54.5)**  **5 (45.5)** | **GG (37)**  **GC+CC (30)** |
| **XRCC3 rs861539** | | | |
| **0.111** | **Reference**  **0.325 (0.078-1.35)** | **3 (27.3)**  **8 (72.7)** | **CC (33)**  **CT+TT (34)** |
| **NBS1 rs1805794** | | | |
| **0.943** | **Reference**  **0.951 (0.249-3.64)** | **4 (36.4)**  **7 (63.6)** | **CC (25)**  **CG+GG (42)** |
| **MRE11 rs569143** | | | |
| **0.391** | **Reference**  **0.538 (0.129-2.34)** | **3 (27.3)**  **8 (72.7)** | **CC(26)**  **CG+GG(41)** |
| **RAD50 rs2299014** | | | |
| **0.108** | **Reference**  **0.343 (0.090-1.31)** | **4 (36.4)**  **7 (63.6)** | **GG (39)**  **GT+TT (28)** |
| **† *Statistically significant difference*** | | | |

| **Association of genetic variants with Dyspnea.** | | | |
| --- | --- | --- | --- |
| ***P.value*** | **OR (95% CI)** | **Dyspnea, n (%)** | **Polymorphism (n)** |
| **RAD51 rs1801320** | | | |
| **0.464** | **Reference**  **0.636 (0.188-2.14)** | **6 (46.2)**  **7 (53.8)** | **GG (37)**  **GC+CC (30)** |
| **XRCC3 rs861539** | | | |
| **0.803** | **Reference**  **0.857 (0.255-2.88)** | **6 (46.2)**  **7 (53.8)** | **CC (33)**  **CT+TT (34)** |
| **NBS1 rs1805794** | | | |
| **0.069** | **Reference**  **0.254 (0.049-1.21)** | **2 (15.4)**  **11 (84.6)** | **CC (25)**  **CG+GG (42)** |
| **MRE11 rs569143** | | | |
| **0.545** | **Reference**  **1.45 (0.499-4.94)** | **6 (46.2)**  **7 (53.8)** | **CC(26)**  **CG+GG(41)** |
| **RAD50 rs2299014** | | | |
| **0.326** | **Reference**  **0.545 (0.161-1.84)** | **6 (46.2)**  **7 (53.8)** | **GG (39)**  **GT+TT (28)** |
| **† *Statistically significant difference*** | | | |

| **Association of genetic variants with Pulmonary edema.** | | | |
| --- | --- | --- | --- |
| ***P.value*** | **OR (95% CI)** | **Pulmonary edema, n (%)** | **Polymorphism (n)** |
| **RAD51 rs1801320** | | | |
| **0.487** | **Reference**  **0.574 (0.118-2.78)** | **3 (42.9)**  **4 (57.1)** | **GG (37)**  **GC+CC (30)** |
| **XRCC3 rs861539** | | | |
| **0.721** | **Reference**  **0.750 (0.154-3.64)** | **3 (42.9)**  **4 (57.1)** | **CC (33)**  **CT+TT (34)** |
| **NBS1 rs1805794** | | | |
| **0.183** | **Reference**  **0.250 (0.028-1.20)** | **1 (14.3)**  **6 (85.7)** | **CC (25)**  **CG+GG (42)** |
| **MRE11 rs569143** | | | |
| **0.557** | **Reference**  **0.600 (0.108-3.34)** | **2 (28.6)**  **5 (71.4)** | **CC(26)**  **CG+GG(41)** |
| **RAD50 rs2299014** | | | |
| **0.384** | **Reference**  **0.500 (0.103-2.43)** | **3 (42.9)**  **4 (57.1)** | **GG (39)**  **GT+TT (28)** |
| **† *Statistically significant difference*** | | | |
